# Engineering Ge Profiles in Si/SiGe Heterostructures for Increased Valley Splitting

**DOI:** 10.1021/acs.nanolett.5c02848

**Published:** 2025-08-12

**Authors:** Lucas E. A. Stehouwer, Merrit P. Losert, Maia Rigot, Davide Degli Esposti, Sara Martí-Sánchez, Maximillian Rimbach-Russ, Jordi Arbiol, Mark Friesen, Giordano Scappucci

**Affiliations:** † QuTech and Kavli Institute of Nanoscience, 2860 Delft University of Technology, Lorentzweg 1, 2628 CJ Delft, The Netherlands; ‡ 5228 University of Wisconsin—Madison, Madison, Wisconsin 53706, United States; ¶ Catalan Institute of Nanoscience and Nanotechnology (ICN2), CSIC, and BIST, 231882 Campus Universitat Autònoma de Barcelona, Bellaterra, 08193 Barcelona, Catalonia, Spain; § Institución Catalana de Investigación y Estudios Avanzados (ICREA), Pg. Lluís Companys 23, 08010 Barcelona, Catalonia, Spain

**Keywords:** heterostructure, mobility, quantum dots, valley splitting, quantum Hall effect

## Abstract

Electron-spin qubits in Si/SiGe quantum wells are limited by the small and variable energy separation of the conduction-band valleys. While sharp quantum-well interfaces are pursued to increase the valley-splitting energy deterministically, here we explore an alternative approach to enhancing the valley splitting on average. We grow increasingly thinner quantum wells with broad interfaces to controllably increase the electron wave function overlap with Ge atoms. Quantum Hall measurements of two-dimensional electron gases reveal a linear correlation between valley splitting and disorder-induced single-particle energy-level broadening, driven by increasing alloy scattering at the Si/SiGe interface. We demonstrate enhanced valley splitting while maintaining respectable electron mobility, indicating a low-disorder electrostatic potential environment. Simulations using experimental Ge concentration profiles predict an average valley splitting in quantum dots that matches the enhancement observed in two-dimensional systems. Our results motivate the experimental realization of quantum-dot spin qubits in these heterostructures.

## Graphical abstract

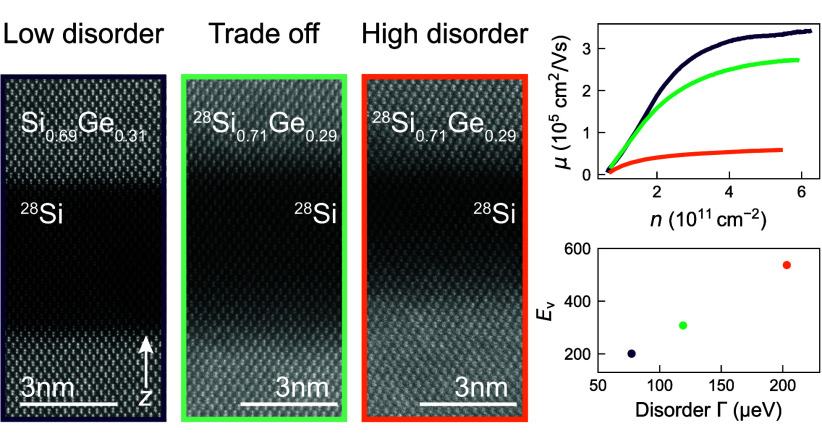

Spin qubit devices in gate-defined Si/SiGe quantum dots have advanced in terms of performance, qubit count, and connectivity. Reproducible one- and two-qubit gate fidelities exceeding 99% have been achieved.^1–3^ Moreover, linear array devices have scaled the number of qubits from 6 ^4^ to 12,^5^ and a two-by-two qubit array has been demonstrated.^6^ Coherent, high-fidelity spin shuttling^7–9^ and cavity-mediated iSWAP oscillations between distant spins^10^ are promising achievements for connectivity beyond nearest neighbor. In addition, the fabrication of Si/SiGe spin qubits in a 300 mm semiconductor manufacturing facility^11^ and the integration of multilevel interconnects with two-dimensional spin qubit arrays^12^ underscore the potential for scalable architectures. Despite this compelling progress, critical material challenges remain in the pursuit of a large-scale quantum computer.

In Si/SiGe heterostructures, a long-standing limitation has been the small and variable energy splitting between the two low-lying conduction-band valleys.^13,14^ In quantum dots, the reported valley-splitting energies vary between tens to hundreds of microelectronvolts,^15–22^ even across a single chip.^17,19,23–25^ This poses a challenge for spin qubits because the increased leakage from the computational two-level Hilbert space affects high-fidelity initialization, control, readout, and shuttling.^26–32^

Recent work combining experiments and theory^33–35^ has established that the atomistic random alloy concentration fluctuations at the Si/SiGe interface (alloy disorder) are accountable for the measured valley-splitting spread in real quantum dots. Furthermore, the valley splitting is expected to be enhanced when the electronic wave function overlaps with more Ge atoms. While proposed strategies like intentionally adding Ge to the Si quantum well promise increased valley splitting,^33,34^ they may also worsen the electrostatic disorder, affecting electron mobility.^20^ However, careful tuning of the Ge concentration profile—through adjustments in the Si quantum-well thickness, interface width, and barrier composition—can strike a delicate balance between achieving high valley splitting and maintaining low disorder.^36^

Here, we engineer the Ge concentration profiles of ^28^Si/^28^SiGe heterostructures to enhance the overlap of the electron wave function with Ge atoms in a tunable way by growing increasingly thin quantum wells with intentionally diffused interfaces. We characterize the Ge concentration profiles by atomic-resolution scanning transmission electron microscopy (STEM), while we measure the mobility and device-averaged valley-splitting energy of the two-dimensional electron gas (2DEG) (*E*_v_) by comprehensive density-dependent magnetotransport. Benchmarking against control heterostructures with sharp interfaces,^36^ we can controllably increase valley splitting by up to a factor of 2. Although we unambiguously observe that higher valley splitting correlates with increased alloy disorder scattering, a beneficial trade-off is achievable between enhanced valley splitting and respectable electron mobility, indicative of low electrostatic disorder. Furthermore, simulations of sample-averaged quantum dot valley-splitting energy (*E*_v_^QD^) based on the experimental Ge concentration profiles, reveal a linear relationship with *E*_v_. This finding provides the first insight into the long-sought connection between valley splitting in the quantum Hall regime and that in quantum dots.

Parts a–c of Figure 1 show atomic-resolution high angle annular dark field (HAADF-)­STEM images of three ^28^Si/^28^SiGe heterostructures (B1–B3) having progressively thinner quantum wells with similarly broad interfaces. As a control, Figure 1d shows ^28^Si/SiGe heterostructure (A) with sharp interfaces, as studied in ref 36. Broad interfaces in heterostructures B1, B2, and B3 result from uninterrupted epitaxy at a temperature of 750 °C using only hydride precursors (^28^SiH_4_ and GeH_4_). In contrast, sharp interfaces in heterostructure A are achieved by growing the SiGe barriers at a lower temperature of 625 °C, enabled by using a different Si precursor (SiH_2_Cl_2_).^33,36^

**Figure 1:**
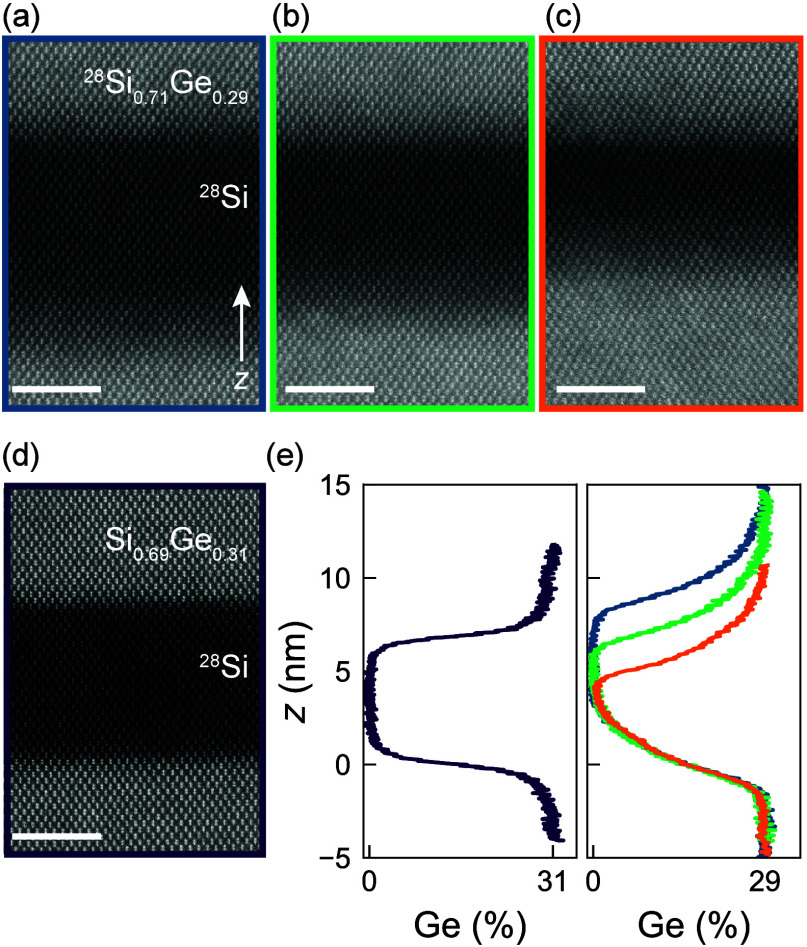
Si/SiGe heterostructures with engineered Ge concentration profiles. (a–c) HAADF-STEM images of ^28^Si/^28^SiGe heterostructures B1 (blue), B2 (green), and B3 (orange), with intentionally diffused quantum-well interfaces. The characterization is performed after H-FET fabrication. The quantum well and surrounding barriers feature isotopically purified ^28^Si. We vary the quantum-well width between B1 and B3 from 9.5 to 5.9 nm (Table 1). (d) HAADF-STEM image of ^28^Si/SiGe control heterostructure A (purple) with sharp interfaces. Only the quantum well is isotopically purified. The scale bar is 3 nm in parts a–d. (e) Ge concentration profiles for heterostructures A (left panel) and B1–B3 (right panel) extracted by combining SIMS data with HAADF-STEM intensity profiles (Figure S1).

In all heterostructures, the quantum well is deposited on a SiGe strain-relaxed buffer and separated from the dielectric interface by a 30 nm SiGe barrier (Section 1). Due to the different gas precursors, heterostructures B1–B3 feature an isotopically enriched barrier with a slightly lower Ge concentration (^28^Si_0.71_Ge_0.29_) compared to heterostructure A (Si_0.69_Ge_0.31_). The small difference in the chemical composition, and therefore the band offset, is confirmed by electrical measurements of the quantum-well saturation charge density,^37,38^ which is smaller in B1–B3 compared to A (Figure S1). In Figure 1e, we show the Ge concentration profiles from A (left panel) and B1–B3 (right panel) extracted from the HAADF-STEM images (Figure S1). The right panel highlights both the reproducibility of the growth process from the overlapping bottom interfaces (*z* = 0 nm) and the control over the quantum-well width. From the concentration profiles, we extract the quantum-well width *w*_QW_ and the width *w*_if_ of the top and bottom interfaces. Table 1 gives a quantitative overview of the extracted parameters. We controllably reduced the quantum-well width between the heterostructures B1 and B3 by adjusting the quantum-well growth time. Notably, the interfaces of heterostructures B1, B2, and B3 are approximately 2.4 times wider than those of heterostructure A.

**Table 1:** Overview of Quantum-Well Metrics^a^

	A	B1	B2	B3
*w*_QW_ (nm)	6.9	9.5	7.8	5.9
*w*_if_^top^ (nm)	1.5	3.7	3.7	3.6
*w*_if_^bottom^ (nm)	1.6	3.3	3.5	3.5

a The quantum-well width *w*_QW_, top interface width *w*_if_^top^, and bottom interface width *w*_if_^bottom^ for heterostructures A, B1, B2, and B3 are given. The quantum-well width is defined as the distance between the top and bottom interfaces, where the Ge concentration reaches 50% of its maximum value. The width of the top and bottom interfaces is defined as the distance over which the Ge concentration rises from 10% to 90% of its maximum value. Uncertainty of the extracted values is assumed to be in the last reported digit. Extracted values are from heterostructures after H-FET fabrication.

We evaluate the electrical properties of the 2DEG in each heterostructure by fabricating Hall-bar-shaped heterostructure field-effect transistors (H-FETs) and performing magnetotransport measurements at 70 mK in a dilution refrigerator equipped with a cryomultiplexer^39^ (Section 4). Parts a and b of Figure 2 show the mobility–density and conductivity–density curves of a representative H-FET for each heterostructure (see Figure S2 for other H-FETs). Heterostructures A, B1, and B2 show similar mobility–density curves, while heterostructure B3 shows a severe suppression of the mobility across the entire density range. In Figure 2c, we show the average extracted mobility for each heterostructure. The maximum mobility decreases from 3.8(4) × 10^5^ cm^2^/(V s) in B1 to 0.58 × 10^5^ cm^2^/(V s) in B3 as the quantum well becomes increasingly thinner. Compared to the control heterostructure A, B1 shows a higher average maximum mobility, which we attribute to the increased growth temperature resulting in decreased background contamination. However, B1 also shows a larger spread across multiple H-FETs, which is indicative of the onset of strain relaxation within the quantum well, creating additional scattering centers from dislocations.^19,36^ Severe reduction of the maximum mobility in B3 is compatible with the presence of Ge throughout the thin quantum well.^40–42^

**Figure 2:**
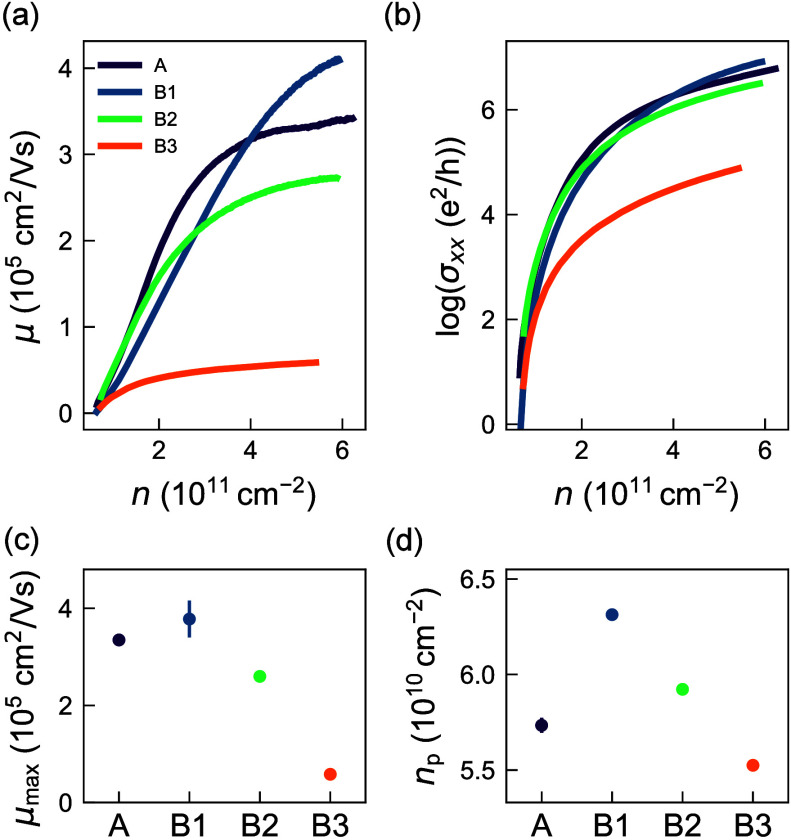
Classical transport measurements of mobility and percolation density. (a) Mobility (μ)–density (*n*) curves for heterostructures A, B1, B2, and B3. (b) Conductivity (σ_*xx*_)–density (*n*) curves of the four heterostructures, from which we fit the percolation density *n*_p_ (Section 4). (c) Average maximum mobility μ_max_ of the four heterostructures (A1 and B1–B3) from measurements of multiple devices. Error bars represent 1 standard deviation around the average. (d) Extracted percolation densities *n*_p_ for the four heterostructures.

In contrast to the observed trend in maximum mobility, we do not observe a strong dependence across different heterostructures of the percolation density *n*_p_ (Figure 2d), obtained by fitting the conductivity curves in Figure 2b (see also Section 4). We find similar low values of around 6.0 × 10^10^ cm^–2^ for all heterostructures within the constraints of the fitting procedure, which is consistent with previous arguments that alloy disorder only weakly affects the scattering rate at low density.^40–42^ This observation suggests that the increased alloy disorder from diffusion of the interfaces does not severely affect the disorder properties of the 2DEG in the low-density regime, which is relevant for quantum dots.

After assessing the electrical properties of the heterostructures, we probe valley splitting in the same H-FETs by performing activation energy measurements in the quantum Hall regime, following ref 43. We focus on the first valley-split energy gap (Δ_1_) at filling factor ν = 1 because this gap is resolved across all heterostructures over a similar range of density *n* and magnetic field *B*, enabling meaningful comparisons. Additionally, we measure the first Zeeman-split gap (Δ_2_) and the first Landau gap (Δ_4_), corresponding to ν = 2 and 4, respectively. Figure 3 illustrates the measurement protocol with data from heterostructure B2, while measurements from all other heterostructures are shown in Figures S3–S5. First, we measure the longitudinal resistivity ρ_*xx*_ at the base temperature as a function of *B*, over a range of fixed densities *n* (Figure 3a). We observe clear Shubnikov–de Haas oscillations, with minima at ν = 1 reaching zero, indicating a well-resolved Δ_1_. For each *n*, we repeat the measurement for different temperatures (*T* = 70–1000 mK) and plot in Figure 3b ρ_*xx*_ as a function of filling factor ν, given by the quantum Hall relationship ν = *nh*/*eB*, where *h* is Planck’s constant and *e* the electron charge. As the inset shows for ν = 1, we observe a thermally activated dependence of the oscillation minima [ρ_*xx*_ ∝ exp­(−Δ/2*k*_B_ *T*)]. For each density, we extract the valley-split, Zeeman split, and Landau mobility gaps (Δ_1_, Δ_2_, and Δ_4_ respectively), plotted in Figure 3c as a function of magnetic field *B*. As in ref 43, we observe striking linear relationships converging to a similar intercept, from which we estimate with confidence the disorder-induced single-particle energy-level broadening^44^ Γ (Figure 3c, side panel) and the valley splitting *E*_v_ = Δ_1_ + Γ.

**Figure 3:**
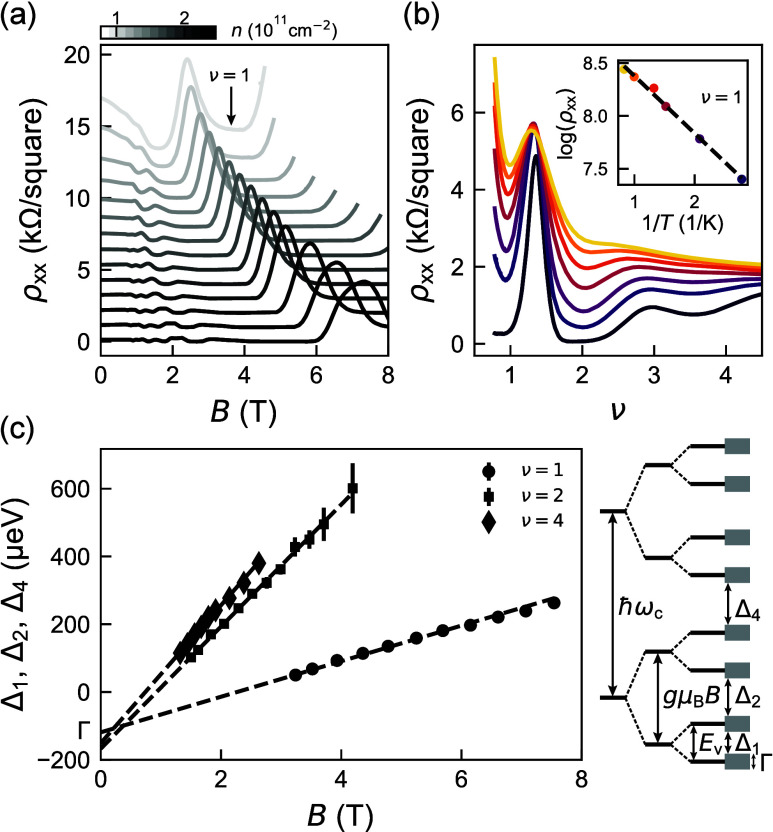
Quantum transport measurements of valley splitting and disorder-induced energy–level broadening. (a) Longitudinal resistivity ρ_*xx*_ of heterostructure B2 as a function of magnetic field *B* over a range of fixed densities *n* between 0.77 × 10^11^ cm^–2^ (light gray) and 2.46 × 10^11^ cm^–2^ (black) (offset for clarity). (b) Magnetotransport measurements at fixed density *n* (here 0.91 × 10^11^ cm^–2^) and at different temperatures *T*, plotted against integer filling factor ν = *nh*/*eB*. Different colors represent different temperatures between 70 mK (dark purple) and 1000 mK (yellow). The inset shows the thermally activated dependence of the oscillation minima [ρ_*xx*_ ∝ exp­(−Δ/2*k*_B_ *T*)] for integer filling factor ν = 1 from which we extract the mobility gap Δ_1_ of the first valley. These measurements are repeated for each density and the analysis for ν = 1, 2, and 4. (c) Mobility gaps of the first valley gap Δ_1_ (ν = 1), the first Zeeman gap Δ_2_ (ν = 2), and the first Landau gap Δ_4_ (ν = 4) as a function of magnetic field *B*. The linear fits (dotted lines) are used for extracting the disorder-induced single-particle energy-level broadening Γ. The side schematic shows the energy-level ladder in the quantum Hall regime, including Γ. Landau levels are split by energy ℏω_c_, the Zeeman levels are split by *g*μ_B_ *B*, and valley levels are split by valley-splitting energy *E*_v_.

Following this systematic classical and quantum transport characterization, we may now investigate the key link between valley splitting and disorder underpinned by the engineered Ge concentration profiles in the different heterostructures. In all heterostructures, *E*_v_ increases linearly with *B* across the investigated range (Figure 4a). Additionally, to access the electrostatic confinement induced by magnetic field *B*, the top *x* axis shows the correspondingly increasing orbital energy, *E*_orb_ = *e*ℏ*B*/2m*, where we use an in-plane effective mass of 0.2*m*_e_ for electrons in Si. Note that the *E*_v_ ∝ *B* relationship was previously observed^43,45^ and attributed^43^ to the stronger electrostatic confinement achieved for a higher density in the quantum Hall edge channel, driven by *B* via the quantum Hall relationship *n* = *eB*/*h* for ν = 1.

**Figure 4:**
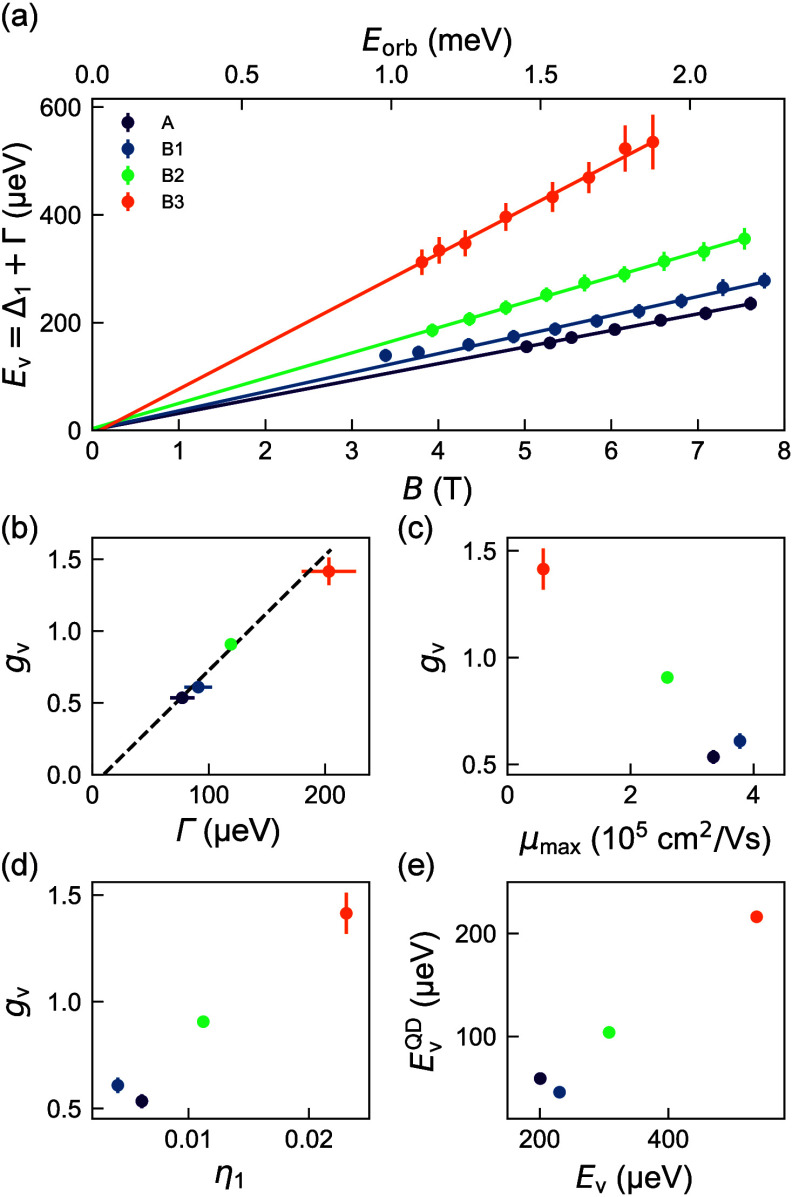
Valley-splitting correlations. (a) Valley-splitting energy *E*_v_ = Δ_1_ + Γ as a function of magnetic field *B* for heterostructures A and B1–B3. The corresponding orbital energy *E*_orb_ is shown in the top *x* axis. (b) Valley *g*-factor *g*_v_, from the slopes of *E*_v_ versus magnetic field *B* in part a, as a function of the corresponding disorder-induced single-particle energy-level broadening Γ. Color coding is as in part a. (c) *g*_*v*_ as a function of maximum mobility μ_max_. Color coding is as in part a. (d) *g*_*v*_ as a function of η_1_, the overlap of the electron wave function with Ge atoms simulated by using the Ge concentration profiles from Figure 1e. (e) Simulated valley-splitting energy in quantum dots *E*_v_^QD^ against the 2DEG valley splitting *E*_v_ from part a evaluated at a magnetic field *B* of 6.5 T. This magnetic field corresponds to an orbital energy of 1.88 meV (see the main text), which is the same orbital energy used for the simulation of *E*_v_^QD^.

Across the explored magnetic field range, we observe a clear trend in Figure 4a: all heterostructures with broad interfaces (B1–B3) show larger valley splitting compared to the control heterostructure A. Moreover, within heterostructures B1–B3, thinner quantum wells achieve larger valley splitting, validating our heterostructure design. To quantify these observations, we extract the valley *g*-factor, *g*_v_ = (1/μ_B_)­(d*E*_v_/d*B*), which represents the rate of change of valley splitting with the magnetic field, normalized to the Bohr magneton μ_B_. Figure 4b shows *g*_v_ against Γ, revealing a striking experimental correlation driven by the increased scattering from alloy disorder. This is further corroborated in Figure 4c by the dependence of *g*_v_ on the maximum mobility. The valley splitting in heterostructures B1–B3 may increase to more than twice the value observed in the control heterostructure A. These clear trends confirm the intuition that increasing valley splitting, which requires breaking translation symmetry, comes at the expense of a more disordered potential landscape,^34,36^ qualified in our experiments either by classical or quantum transport measurements.

Next, we investigate the atomistic origin of the increased valley splitting, *E*_v_, and provide a prognosis for potential gains for valley splitting in quantum dots. To this end, we calculate for each heterostructure the parameter η_1_ (Section 5), which quantifies the overlap of the electron wave function with Ge atoms, and simulate the quantum-dot valley-splitting distributions and their mean value *E*_v_^QD^. We use the extracted Ge concentration profiles of Figure 1e as an experimental input for the simulation methods in ref 34. As shown in Figure 4d, unambiguous correlation between *g*_v_ and η_1_ was found, suggesting that the larger *E*_v_ measured in the 2DEG correlates with the increased overlap of the electron wave function with Ge atoms, promoted in our experiments by thinner quantum wells with broad interfaces. This finding (*E*_v_ ∝ η_1_) mirrors the theoretical predictions for average valley splitting in alloy-disorder-dominated quantum dots (*E*_v_^QD^ ∝ η_1_ (Section 5 and refs 33 and 34). As a consequence, the plot in Figure 4e of simulated *E*_v_^QD^ against experimentally measured *E*_v_ also shows a linear relationship. Here, we choose to simulate *E*_v_^QD^ at an orbital energy *E*_orb_ of 1.88 meV, which is on par with measured values in quantum dots and corresponds to an experimentally accessible magnetic field of 6.5 T for the evaluation of *E*_v_, as shown in Figure 4a. Considering the same orbital energy ensures a meaningful comparison between the experimentally informed simulation of *E*_v_^QD^ and the measured *E*_v_, although we note that confinement in qubit experiments is imposed electrostatically via top gates, while magnetic fields are typically applied in-plane. While offering a first insight into the relationship between the two metrics, on the basis of these results, we predict that heterostructures B1–B3 could support, on average, increased valley splitting in quantum dots, which is proxied by valley splitting measured in the quantum Hall regime.

In summary, we have engineered ^28^Si/^28^SiGe heterostructures to enhance the overlap of the electron wave function with Ge atoms by growing increasingly thin quantum wells with intentionally diffused interfaces. Our comprehensive study unveils unambiguously a correlation between disorder in the 2DEG, driven by random alloy scattering and measured with classical and quantum transport, and valley splitting, measured in the quantum Hall regime. Valley splitting is increased but so is alloy disorder. On the basis of simulations that take into account the experimental Ge concentration profile, we identify the overlap of the electron wave function with Ge atoms as the likely cause of this connection, which also propagates to calculated average values of valley-splitting distributions in quantum dots.

Compared to control samples with sharp interfaces in ref 36, we show that a quantum well with much broader interfaces (≃3.6 nm) and similar width (≃7.8 nm) offers an excellent trade-off, featuring a 1.8× valley-splitting increase, while still having respectable mobility [>2 × 10^5^ cm^2^/(V s)] and low percolation density (<6 × 10^10^ cm^–2^). In contrast, thinner or thicker quantum wells either significantly degrade mobility or yield only marginal improvements in valley splitting. Future statistical studies of valley splitting in quantum dots fabricated on these new generations of heterostructures are required to confirm the valley-splitting increase and assess the impact of alloy disorder in the formation of spurious quantum dots.

## Supplementary Material



## Data Availability

The data sets supporting the findings of this study are openly available in 4TU Research Data at 10.4121/ebcf5563-628e-479c-9e0d-d5094ebb9c27.

## Notes

Growth conditions of heterostructures A, B1, B2, and B3, STEM sample preparation and characterization, extraction of Ge concentration profiles from STEM and SIMS, description of H-FET measurements, valley-splitting simulations based on Ge concentration profiles of the quantum wells, SIMS data detailing SiGe compositions, extraction of the maximum electron density of each heterostructure, mobility–density results of multiple H-FETs for each heterostructure, magnetotransport measurements for heterostructures A, B1, and B3, and distribution of simulated valley-splitting energies in quantum dots (PDF)
