# Supplementary material for: Engineering Ge Profiles in Si/SiGe Heterostructures for Increased Valley Splitting
Source: Nano Lett. 2025 Aug 12;25(34):12892–8. doi: 10.1021/acs.nanolett.5c02848 (PMC12395482; doi:10.1021/acs.nanolett.5c02848)
Supplement: Supplementary file 1 [file nl5c02848_si_001.pdf]

Supporting information to: Engineering Ge profiles in Si/SiGe heterostructures for increased valley splitting

Lucas E. A. Stehouwer,[†] Merrit P. Losert,[‡] Maia Rigot,[†] Davide Degli Esposti,[†] Sara Martí-Sánchez,[¶] Maximillian Rimbach-Russ,[†] Jordi Arbiol,[¶,§] Mark Friesen,[‡] and Giordano Scappucci*,[†]

†*QuTech and Kavli Institute of Nanoscience, Delft University of Technology, Lorentzweg 1, 2628 CJ Delft, The Netherlands*

‡*University of Wisconsin-Madison, Madison 53706 WI, USA*

¶*Catalan Institute of Nanoscience and Nanotechnology (ICN2), CSIC and BIST, Campus UAB, Bellaterra, 08193 Barcelona, Catalonia, Spain*

§*ICREA, Pg. Lluís Companys 23, 08010 Barcelona, Catalonia, Spain*

E-mail: g.scappucci@tudelft.nl

1 Heterostructure growth

We grow the Si/SiGe heterostructures in an ASM Epsilon 2000 reduced pressure chemical vapour deposition reactor (RP-CVD). Each heterostructure is grown on a 100 mm n-type Si(001) wafers and begins with a step graded $Si_{1-x}Ge_x$ virtual substrate (VS) grown at 750 °C where the Ge content is graded to a final percentage of 31% in four steps (x = 0.07, 0.14, 0.21, 0.31). For heterostructure A, we subsequently lower the growth temperature to 625 °C to grow a $Si_{0.69}Ge_{0.31}$ strain-relaxed buffer (SRB) using dichlorosilane and germane as precursor gases. We then grow the isotopically purified ^{28}Si quantum well (800 ppm residual ^{29}Si isotopes[1–3]) using silane at a temperature of 750 °C, followed by a 30 nm thick $Si_{0.69}Ge_{0.31}$ spacer layer using the same growth conditions as the SRB. Finally, we grow a thin ^{28}Si capping layer using silane at a temperature of 750 °C.

For heterostructures B1-B3 we use use the same step graded VS as in A but subsequently continue the growth of the $^{28}Si_{0.71}Ge_{0.29}$ SRB, the ^{28}Si quantum well, the 30 nm $^{28}Si_{0.71}Ge_{0.29}$ spacer, and ^{28}Si capping layer at a growth temperature of 750 °C using silane and germane as precursor gases.

For the fabrication of the Hall bars we use the identical procedure as described in the Methods section of Ref. [4] .

2 (S)TEM

For structural characterization with (S)TEM, we prepared lamella cross-sections of the quantum well heterostructures by using a Focused Ion Beam (Helios 5UX). Atomically resolved HAADF-STEM data was acquired in a double-corrected Thermo Fisher Spectra 300 microscope operated at 300 kV.

3 Estimation of Ge concentration profiles

The Ge concentration profiles presented in Fig. 1(e) are estimated by combining HAADF-STEM data and secondary ion mass spectroscopy (SIMS) data. The atomic resolution HAADF-STEM images presented in Fig. 1 provide a high spatial accuracy which we make use of by extracting an intensity profile from them. SIMS provides information about the silicon-germanium composition in the barrier layers of the heterostructures. We use the SiGe composition data (see Supplementary Fig. S1) of the SiGe barrier layers to rescale the HAADF-STEM intensity profiles to the Ge concentration profiles.

4 H-FET measurements

Characterisation of the Hall bars is done in a Leiden cryogenics refrigerator with a base temperature of 70 mK. We apply a 100 μV source-drain bias and measure the current I_{sd}, longitudinal voltage V_{xx} and transverse voltage V_{xy} as a function of gate voltage V_{g} and perpendicular magnetic field B using four-probe low-frequency lock-in techniques. We calculate longitudinal ρ_{xx} and transverse ρ_{xy} resistivity from which we extract the Hall density n at low magnetic fields using $\rho_{\mathrm{xy}} = B/en$, where e is the electron charge. Mobility is found using $\mu = 1/ne\rho_{\mathrm{xx}}$. At $B = 0$ we calculate the conductivity $\sigma_{\mathrm{xx}} = 1/\rho_{\mathrm{xx}}$ and extract the percolation density n_{p} using fitting formula $\sigma_{\mathrm{xx}} \propto (n - n_{\mathrm{p}})^{1.31}$. We measure a total of 9, 10, 10, and 8 Hall bars for heterostructures A, B1, B2, and B3 respectively.

5 Valley splitting simulations

In Refs.,[5,6] it was demonstrated that the expected valley splitting of a disorder-dominated quantum dot is given by

$$\bar{E}_{\mathrm{v}} = \frac{a_0^2 \Delta E_c}{8 a_{\mathrm{dot}} \Delta G} \sqrt{\sum_l \psi_{\mathrm{env}}^4(z_l) G_l (1 - G_l)} \tag{1}$$

where $a_0 = 0.543$ nm is the Si lattice constant, ΔE_c is the conduction band offset, $a_{\mathrm{dot}} = \sqrt{\hbar^2/m_t E_{\mathrm{orb}}}$ is the dot radius, $\Delta G \approx 0.3$ is the Ge concentration offset between the quantum well and SiGe barriers, $\psi_{\mathrm{env}}(z)$ is an envelope function, G_l is the Ge concentration at atomic layer l, and the sum is taken over all atomic layers in the heterostructure. We use Eq. (1) to compute the average quantum dot valley splittings $E_{\mathrm{v}}^{\mathrm{QD}}$ reported in Fig. 4 of the main text. In these calculations, we determine ψ_{env} by diagonalizing a virtual crystal Hamiltonian

$$H_{\mathrm{vc}} = -\frac{\hbar^2}{2m_l}\partial_z^2 + U_{\mathrm{qw}} + U_z, \tag{2}$$

where $m_l = 0.916 m_e$ is the longitudinal effective mass in Si. The quantum well potential is given by

$$U_{\mathrm{qw}}(z) = \Delta E_c \frac{G(z) - G_{\mathrm{min}}}{\Delta G}, \tag{3}$$

where G_{min} is the minimum Ge concentration in the quantum well, determined from the estimated Ge concentration profiles described in Sec. 3, and $G(z)$ are shown in Fig. 1. The conduction band offset ΔE_c is computed following Ref.[5]. The electrostatic potential is given by $U_z = -eE_z z$ for constant vertical electric field $E_z = 1$ mV/nm. While somewhat arbitrary, this E_z is consistent with prior analysis of quantum dot devices in Si/SiGe.[7] Additionally, prior simulations have indicated only a minor dependence of $E_{\mathrm{v}}^{\mathrm{QD}}$ on E_z for E_z between 0 and 5 mV/nm,[5,8] especially for the narrow quantum wells considered in this work. Thus, we do not expect this choice to significantly impact our results.

Examining Eq. 1, we can define the dimensionless quantity

$$\eta_1 = \Delta z \sqrt{\sum_l \psi_{\mathrm{env}}^4(z_l) G_l (1 - G_l)}, \tag{4}$$

where $\Delta z = a_0/4$ is the spacing between layers in the heterostructure, such that $\bar{E}_{\mathrm{v}} \propto \eta_1$. This quantity captures the overlap of the quantum dot wavefunction into regions with non-zero Ge concentration and is thus a metric for the impact of alloy disorder on the system.

To determine ψ_{env} for a Hall bar, we self-consistently solve the Schrödinger and Poisson equations. The Schrödinger-Poisson virtual crystal Hamiltonian is

$$H_{\mathrm{vc}}^{\mathrm{sp}} = -\frac{\hbar^2}{2m_l}\partial_z^2 + U_{\mathrm{qw}} - e\phi(z) \tag{5}$$

where the electrostatic potential $\phi(z)$ is determined from Poisson's equation,

$$\partial_z^2\phi(z) = -\frac{\rho(z)}{\epsilon} = -\frac{|\psi_{\mathrm{env}}(z)|^2}{\epsilon} \tag{6}$$

where $\epsilon = \epsilon_{\mathrm{Si}}\epsilon_0$, and $\epsilon_{\mathrm{Si}} = 11.4$ is the dielectric constant of Si. To bound these simulations, we choose a total electron density of 1.5×10^{11} cm^{-2}, chosen to be within the range of densities observed in the experiment, and we enforce $\partial_z\phi(z) = 0$ far below the quantum well. Again, since the overlap of the wavefunction with high-Ge layers is only weakly dependent on the vertical field in narrow quantum wells, we do not expect this choice to significantly impact our results.

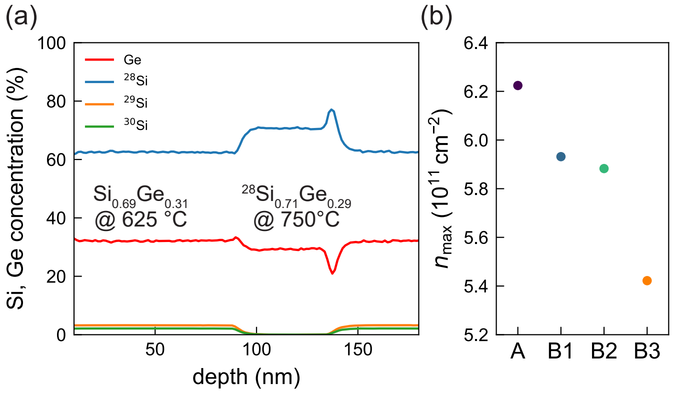

Figure S1: **Chemical composition and saturation density.** (a) Secondary ion mass spectroscopy (SIMS) of a test structure for the growth of SiGe with isotopically enriched ^{28}Si. The test structure consists of a layer of $^{28}Si_{0.71}Ge_{0.29}$ grown at 750 °C sandwiched between two layers of all natural $Si_{0.69}Ge_{0.31}$ grown at 625 °C. We use the same growth conditions at 750 °C of $^{28}Si_{0.71}Ge_{0.29}$ for the SiGe barrier layers in heterostructures B1, B2, and B3 of the main text. Heterostructure A uses the growth conditions of the natural $Si_{0.69}Ge_{0.31}$ at 625 °C. (b) Saturation density n_{max} for the different heterostructures. We find that B1–B3 show a lower maximum density compared to A, which we attribute to the reduced Ge composition of the barrier layers in these heterostructures. B3 shows an even lower maximum density which most likely is the result of having Ge throughout the entire quantum well in this heterostructure.

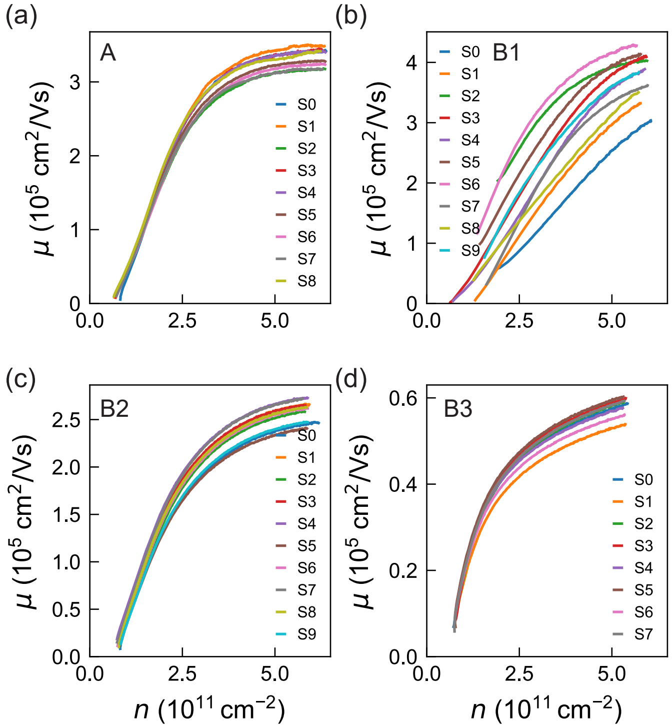

Figure S2: **Mobility-density characterisation.** (a)-(d) We present the mobility-density results measured across multiple H-FETs (S0-S9) on each heterostructure (A, B1-B3). Heterostructures A, B2, and B3 show uniform mobility-density curves across multiple H-FETs. B1 shows relatively less uniformity, which we attribute to possible strain-relaxation of the quantum well due to a combination of increased growth temperature of the SiGe barrier layers and a comparatively thick ^{28}Si quantum well of 9.5 nm.

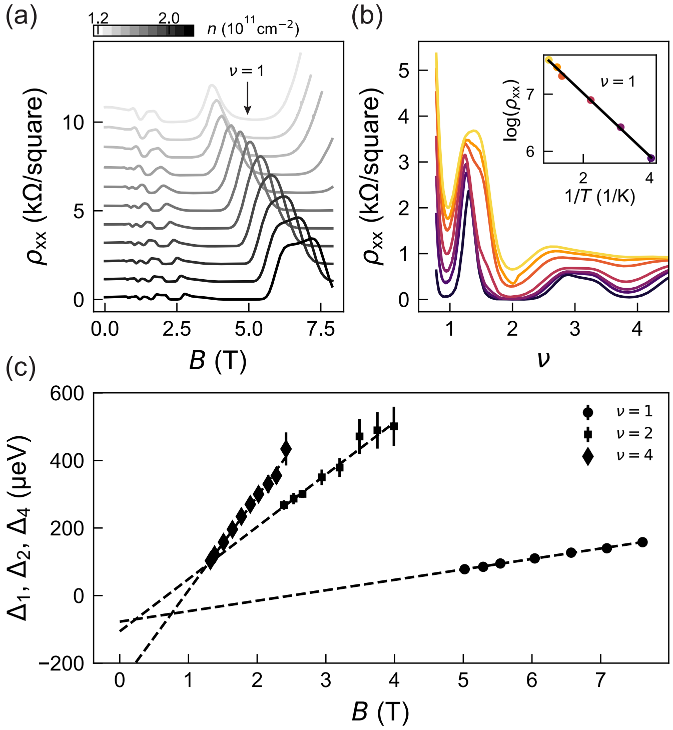

Figure S3: **Magnetotransport measurements for heterostructure A.** (a) We measure longitudinal resistivity ρ_{xx} as a function of magnetic field B for fixed densities n represented by the different gray colours. (b) For each density n (here 1.28×10^{11} cm^{-2}) we repeat the magnetotransport measurements at different temperatures T (70-1000 mK) and plot them against integer filling factor $\nu = nh/eB$. At each integer filling factor ν we extract the corresponding value of ρ_{xx} and find a thermally activated dependency given by $\rho_{\mathrm{xx}} \propto \exp -\Delta/2k_{\mathrm{B}}T$ (see inset), from which we extract the mobility gap Δ. Here we extract Δ_{v} for $\nu = 1$ corresponding to the first valley gap. (c) We plot the mobility gaps of the first valley gap Δ_{v} ($\nu = 1$), the first Zeeman gap Δ_{z} ($\nu = 2$), and the first Landau gap Δ_{L} ($\nu = 4$) as function of magnetic field B. From a linear fit (dotted line) we extract the Landau level broadening induced disorder Γ.

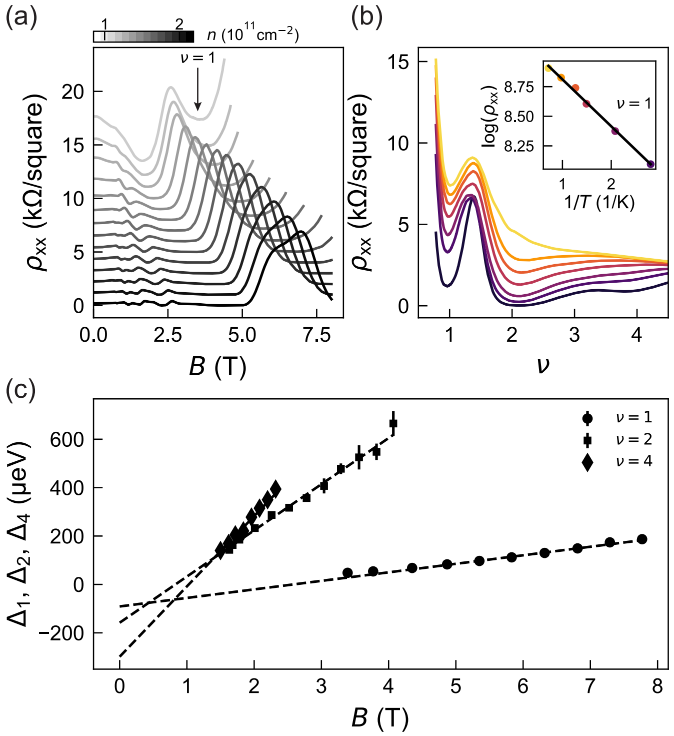

Figure S4: **Magnetotransport measurements for heterostructure B1.** (a) We measure longitudinal resistivity ρ_{xx} as a function of magnetic field B for fixed densities n represented by the different gray colours. (b) For each density n (here 1.05×10^{11} cm^{-2}) we repeat the magnetotransport measurements at different temperatures T (70-1000 mK) and plot them against integer filling factor $\nu = nh/eB$. At each integer filling factor ν we extract the corresponding value of ρ_{xx} and find a thermally activated dependency given by $\rho_{\mathrm{xx}} \propto \exp -\Delta/2k_{\mathrm{B}}T$ (see inset), from which we extract the mobility gap Δ. Here we extract Δ_{v} for $\nu = 1$ corresponding to the first valley gap. (c) We plot the mobility gaps of the first valley gap Δ_{v} ($\nu = 1$), the first Zeeman gap Δ_{z} ($\nu = 2$), and the first Landau gap Δ_{L} ($\nu = 4$) as function of magnetic field B. From a linear fit (dotted line) we extract the Landau level broadening induced disorder Γ.

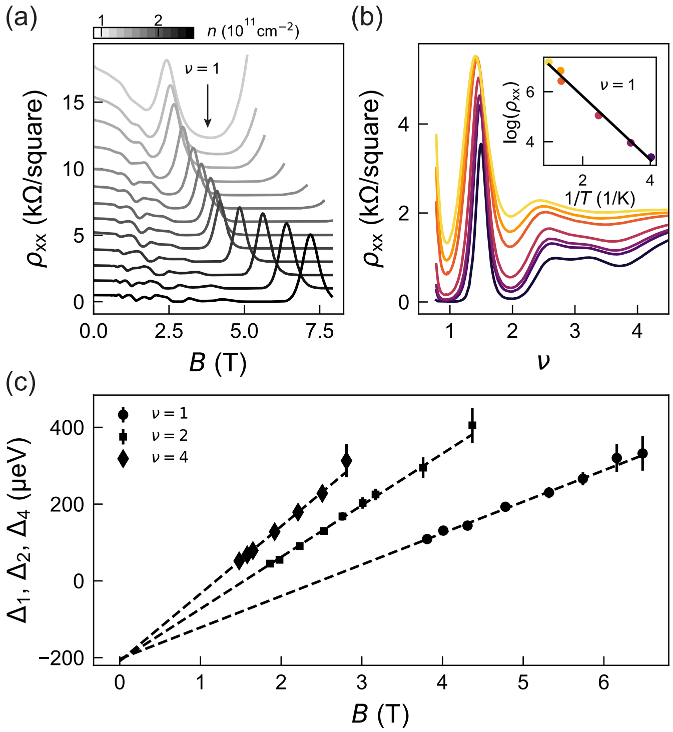

Figure S5: **Magnetotransport measurements for heterostructure B3.** (a) We measure longitudinal resistivity ρ_{xx} as a function of magnetic field B for fixed densities n represented by the different gray colours. (b) For each density n (here 1.21×10^{11} cm^{-2}) we repeat the magnetotransport measurements at different temperatures T (70-1000 mK) and plot them against integer filling factor $\nu = nh/eB$. At each integer filling factor ν we extract the corresponding value of ρ_{xx} and find a thermally activated dependency given by $\rho_{\mathrm{xx}} \propto \exp -\Delta/2k_{\mathrm{B}}T$ (see inset), from which we extract the mobility gap Δ. Here we extract Δ_{v} for $\nu = 1$ corresponding to the first valley gap. (c) We plot the mobility gaps of the first valley gap Δ_{v} ($\nu = 1$), the first Zeeman gap Δ_{z} ($\nu = 2$), and the first Landau gap Δ_{L} ($\nu = 4$) as function of magnetic field B. From a linear fit (dotted line) we extract the Landau level broadening induced disorder Γ.

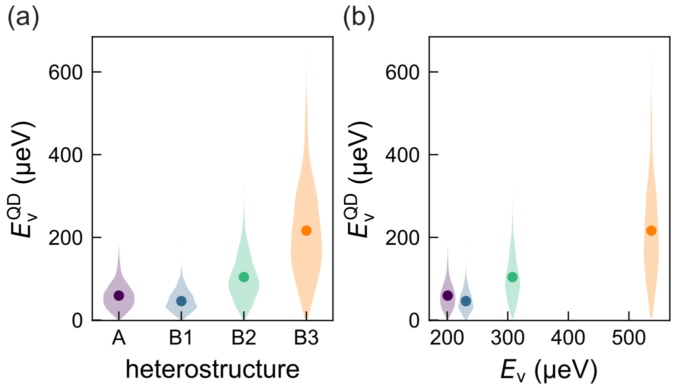

Figure S6: **Distribution of simulated valley splitting energies in quantum dots.** (a) Violin plots showing the distribution of simulated valley splitting energies in quantum dots as a function of heterostructure. Dots show the average valley splitting energy as reported in Fig. 4(e) of the main text. Simulations are performed at an orbital energy of 1.88 meV. (b) Violin plots showing the distribution of simulated valley splitting energies in quantum dots as a function measured valley splitting energy E_v as measured with H-FETs, extracted at a magnetic field of 6.5 T corresponding to an orbital energy of 1.88 meV.

References

(1) Xue, X. et al. CMOS-based cryogenic control of silicon quantum circuits. *Nature* **2021**, *593*, 205–210.

(2) Sabbagh, D. et al. Quantum Transport Properties of Industrial Si28/SiO228. *Physical Review Applied* **2019**, *12*, 014013.

(3) Xue, X.; Russ, M.; Samkharadze, N.; Undseth, B.; Sammak, A.; Scappucci, G.; Vandersypen, L. M. K. Quantum logic with spin qubits crossing the surface code threshold. *Nature* **2022**, *601*, 343–347.

(4) Degli Esposti, D. et al. Low disorder and high valley splitting in silicon. *npj Quantum Information* **2024**, *10*, 32.

(5) Paquelet Wuetz, B. et al. Atomic fluctuations lifting the energy degeneracy in Si/SiGe quantum dots. *Nature Communications* **2022**, *13*, 7730.

(6) Losert, M. P.; Eriksson, M. A.; Joynt, R.; Rahman, R.; Scappucci, G.; Coppersmith, S. N.; Friesen, M. Practical strategies for enhancing the valley splitting in Si/SiGe quantum wells. *Phys. Rev. B* **2023**, *108*, 125405.

(7) Hollmann, A.; Struck, T.; Langrock, V.; Schmidbauer, A.; Schauer, F.; Leonhardt, T.; Sawano, K.; Riemann, H.; Abrosimov, N. V.; Bougeard, D.; Schreiber, L. R. Large, Tunable Valley Splitting and Single-Spin Relaxation Mechanisms in a Si/Si_xGe_{1-x} Quantum Dot. *Physical Review Applied* **2020**, *13*, 034068.

(8) Losert, M. P.; Oberländer, M.; Teske, J. D.; Volmer, M.; Schreiber, L. R.; Bluhm, H.; Coppersmith, S.; Friesen, M. Strategies for Enhancing Spin-Shuttling Fidelities in Si/SiGe Quantum Wells with Random-Alloy Disorder. *PRX Quantum* **2024**, *5*, 040322.
